# Neuroprosthetics: from sensorimotor to cognitive disorders

**DOI:** 10.1038/s42003-022-04390-w

**Published:** 2023-01-06

**Authors:** Ankur Gupta, Nikolaos Vardalakis, Fabien B. Wagner

**Affiliations:** Univ. Bordeaux, CNRS, IMN, UMR 5293, F-33000 Bordeaux, France

**Keywords:** Brain-machine interface, Learning and memory, Diseases of the nervous system

## Abstract

Neuroprosthetics is a multidisciplinary field at the interface between neurosciences and biomedical engineering, which aims at replacing or modulating parts of the nervous system that get disrupted in neurological disorders or after injury. Although neuroprostheses have steadily evolved over the past 60 years in the field of sensory and motor disorders, their application to higher-order cognitive functions is still at a relatively preliminary stage. Nevertheless, a recent series of proof-of-concept studies suggest that electrical neuromodulation strategies might also be useful in alleviating some cognitive and memory deficits, in particular in the context of dementia. Here, we review the evolution of neuroprosthetics from sensorimotor to cognitive disorders, highlighting important common principles such as the need for neuroprosthetic systems that enable multisite bidirectional interactions with the nervous system.

## Abstract (web summary)

This state-of-the-art Review highlights the evolution of neuromodulation and neuroprosthetic systems from sensorimotor to cognitive disorders.

## Introduction

Neural oscillations are ubiquitous throughout the nervous system, subserving sensory, motor, and cognitive functions in the brain and the spinal cord^1^. These oscillations are often disrupted in neurodegenerative and neuropsychiatric disorders^2^, after stroke^3^, traumatic brain injury^4^, or spinal cord injury (SCI)^5^. Despite different underlying mechanisms, abnormal or even abolished neural oscillations seem to be a hallmark of neurological disorders and to play a causative role in their behavioral symptoms^6^. In recent years, bioelectronic devices that interface with the nervous system and affect or reestablish these oscillations have become an alternative to pharmacological treatments, opening a new era of medicine consisting of electroceuticals, Brain-Computer or Brain-Machine Interfaces (BCI/BMI), and neuroprostheses^7–10^.

These bioelectronic devices can now treat some motor symptoms of Parkinson’s disease (PD)^11^ or restore motor function after SCI^12–14^. However, cognitive disorders still elude this new therapeutic modality, due to the intrinsic complexity of the neural mechanisms underlying cognition and to technological challenges for modulating the associated networks. Whether they result from brain injury or neurodegenerative diseases such as Alzheimer’s disease (AD), cognitive disorders do not yet have efficient pharmacological treatments, represent a major public health issue, and could potentially benefit from neuromodulation strategies^15^.

Here, we review the current state of the art on neuromodulation and neuroprosthetic approaches for sensorimotor and cognitive disorders, with a focus on invasive neurotechnologies tested in non-human primates (NHP) and humans. We first introduce the concept of motor neuroprostheses for motor impairments resulting from stroke or SCI, and somatosensory neuroprostheses for providing sensory feedback after limb amputation or paralysis. Next, we present deep brain stimulation (DBS) for motor and cognitive symptoms of PD. We then discuss the neural oscillations that characterize cognitive functions, especially learning and memory, and their alterations in memory disorders. This is followed by a state of the art on neuromodulation approaches for AD and other memory impairments. Finally, we present the enticing hypothesis that cognitive deficits would particularly benefit from the development of a new generation of neuroprosthetic systems that target large-scale brain network oscillations and facilitate the associated neurological functions.

### Motor neuroprostheses

Motor neuroprostheses refer to a particular type of neuroprostheses that aim at restoring motor function by electrical stimulation of the structures involved in the generation of movement (muscles, peripheral nerves, spinal cord, or brain), after neuromotor disorders such as stroke or SCI^16^. The very first motor neuroprosthesis was a peroneal nerve stimulator invented in 1961 by Liberson and colleagues to treat foot drop after hemiplegia^17^. The term neuroprosthesis itself was first coined in the scientific literature in 1971 to refer to an intraspinal implant that allowed bladder voiding after paraplegia^18^. Since then, the definition of motor neuroprostheses has also been extended to technologies that extract motor commands from brain signals to control external devices, also called BCI or BMI (e.g. refs. ^19,20^). In this section, we briefly review the different types of motor neuroprostheses, ranging from neurostimulation of muscles, peripheral nerves, and spinal cord, to implantable BMIs.

#### Functional electrical stimulation (FES)

FES is a clinically approved neurostimulation technology that activates the efferent axons innervating specific muscles to produce a desired movement^21^. The stimulation can be delivered in the vicinity of the targeted muscle or to a motor nerve innervating it, in which case it is called peripheral nerve stimulation (PNS), using either non-invasive, percutaneous, or fully implanted electrodes. These electrodes are in turn connected to an electrical stimulator, which can typically control up to 16 independent channels. Such stimulation systems may simply be used to build up muscle strength, often referred to as NeuroMuscular Electrical Stimulation, or they can assist in functional tasks. Moreover, FES can serve as an assistive technology, reducing impairment in the execution of a given movement, or as part of a rehabilitation therapy that may lead to neuroplasticity and functional improvements, depending on the underlying disorder and its severity^21^.

FES has been applied over the past 60 years to both upper- and lower-limb motor tasks, such as standing, walking, reaching, and grasping^21,22^. Several FDA-approved or CE-marked systems are now commercially available for these applications. Clinically, FES has been mostly used for hemiplegia resulting from stroke, for example, as a foot-drop stimulator^17^, and for the rehabilitation of upper-limb motor function^23^. In the context of SCI, FES has also been extensively tested in research studies^24,25^ but has not yet become standard clinical practice. Overall, FES systems represent the main class of neuroprostheses that have been clinically accepted, but they remain mostly restricted to post-stroke motor rehabilitation. However, FES suffers from a conceptual limitation as it recruits motor axons in a non-physiological order. The large-diameter motor axons that produce large forces but are not resistant to fatigue are indeed recruited first^26–28^, which makes it challenging for FES to sustain large forces during extended periods of time. To circumvent this limitation, methods that target sensory afferents and activate reflex circuits within the spinal cord are desirable.

#### Spinal cord stimulation (SCS)

Well established as a treatment for chronic pain, SCS has recently received significant attention for its applications in motor control^29^. Epidural SCS can be delivered via percutaneous or fully implanted leads containing up to 16 electrodes placed in the posterior epidural space, which are then connected to an external or an implantable pulse generator (IPG). The mechanisms underlying its motor effects involve the activation of large-diameter afferent fibers located in the posterior roots, which in turn recruit motoneuron pools at the innervated spinal segment^30,31^.

The first observation that SCS may be used as a motor neuroprosthesis after SCI was made by Barolat and colleagues in 1986^32^. They showed that one subject with incomplete SCI (i.e. with residual sensory or motor fibers crossing the injury site) regained voluntary motor control with SCS after several months of stimulation. A decade later, Dimitrijevic and colleagues demonstrated that SCS delivered over the lumbar spinal cord could produce rhythmic electromyographic responses and flexion-extension leg movements similar to stepping^33^. These results were obtained in six subjects with complete SCI (i.e. with no residual fiber across the injury) and provided indirect evidence for the existence of a Central Pattern Generator (CPG) in humans^34^. Later studies showed that varying stimulation frequency could also produce a bilateral extension of the lower limbs^35,36^.

The first combination of SCS with locomotor training was performed in 2002 in a subject with incomplete SCI placed in a partial weight-bearing system^37–39^. SCS led to an immediate facilitation of walking, which further improved during training but was not maintained without SCS. The combination of SCS and neurorehabilitation was then revived in 2011 by Harkema and colleagues, who showed that SCS allowed independent, full weight-bearing standing after 80 sessions of intensive training in one subject with complete SCI^40^. Importantly, this study re-discovered that SCS can enable voluntary movements of paralyzed muscles, as first observed by Barolat in 1986. Similar results were then confirmed in three more participants^41,42^ and later replicated at the Mayo Clinic^43^. One subject continued to train with SCS for 3.7 years, which led to a partial recovery of voluntary leg movements without SCS, indicating that SCS has the potential to trigger neuroplasticity mechanisms^44^.

A milestone for the application of SCS in people with SCI was reached in 2018. For the first time, three independent groups demonstrated in a total of six subjects that SCS combined with intensive rehabilitation, could enable independent overground walking^13,45,46^. At the Kentucky SCI Center, four participants with motor-complete SCI performed training sessions for standing, body-weight-supported treadmill stepping and overground walking, all with continuous SCS^45^. When using SCS, all participants achieved assisted standing and improved trunk stability while sitting. Moreover, the two participants with motor-complete, sensory-incomplete SCI gained the ability to walk overground with continuous SCS and assistive devices after a period of 15 and 85 weeks. Similarly, at the Mayo Clinic, an individual with chronic motor- and sensory-complete SCI was trained to perform step-like movements with SCS in a side-lying position^43^ and then on a treadmill and overground^46^. After 43 weeks of training and with continuous SCS, this participant was able to stand, step on a treadmill and walk overground with a walker and manual assistance for hip stability.

In parallel, Courtine and Bloch’s team in Lausanne pioneered a new paradigm termed spatiotemporal SCS^13^. Based on prior preclinical work^47,48^, they leveraged an IPG with real-time control capabilities to develop a stimulation protocol that alternated between the swing, weight acceptance, and propulsion phases of the gait cycle. These functionalities were targeted by spatially-specific electrode configurations, which were triggered either at a pre-defined pace or in real-time by residual kinematic events. Spatiotemporal SCS led to an immediate facilitation of walking and a long-term recovery of motor function within 6 months of training in three subjects with chronic incomplete SCI^13^. This approach was recently expanded to a total of nine participants^49^, including three patients with motor-complete SCI implanted with a new electrode array tailored for both leg and trunk motor functions^14^. Overall, these results have created a surge of interest to now integrate SCS into rehabilitation protocols after SCI.

#### Brain-controlled motor neuroprostheses

Both FES and SCS are neuroprostheses that can be controlled manually or by external kinematic events through external sensors that detect specific state changes. These state changes can also be extracted directly from brain signals through the use of BCI/BMI technologies. Such technologies record brain activity either non-invasively by electroencephalography (not discussed here), or invasively by electrocorticographic (ECoG) grids placed above or below the dura mater, or directly inside the brain by intracranial electrodes^50^.

Intracortical BMIs take their roots in early studies by Georgopoulos and colleagues in 1982, who showed that neurons in the motor cortex of NHPs performing a reaching task are tuned to the direction of movement^51^. With the advent of high-density microwire or microelectrode arrays (MEA), such as the Utah array, neurophysiologists then became able to record tens or hundreds of cells simultaneously from motor cortices. In the early 2000s, three groups led by Nicolelis, Donoghue and Schwarz, demonstrated in NHPs that these intracortical MEAs, combined with signal processing and machine learning, allowed to decode in real-time the intended hand trajectory and to control artificial devices such as a computer cursor or robotic arms^20,52–54^.

A few years later, Hochberg and colleagues translated these findings into a tetraplegic individual implanted with a 96-channel MEA in the hand area of the primary motor cortex^55^. Neural signals still showed movement-related modulation of spiking activity even after several years of paralysis. Moreover, algorithms to decode 2D movement intentions and discrete neural states enabled the participant to open and close a prosthetic hand, control a computer cursor and operate various software. The same group later demonstrated that two additional subjects could use this technology to control a robotic arm with three degrees of freedom (DOF) for the end-point trajectory and discrete states for hand grasping^56^. In parallel, Collinger and colleagues trained a participant implanted with two 96-channel MEAs in the motor cortex to control a robotic arm with up to seven DOF^57^. To control more complex movements that require bilateral coordination, intracortical BMIs can also be combined with additional control strategies, as demonstrated by Tenore’s work on the control of two prosthetic limbs (up to 12 DOF) for bimanual self-feeding^58^. In a recent application, Shenoy’s group expanded intracortical BMIs to the decoding of handwriting (31 characters), which allowed one subject to type sentences at speeds comparable with typical smartphone typing speeds^59^.

Less invasive than intracortical MEAs, ECoG implants consist of grids or strips of electrodes placed over the cortical surface or the dura mater, and are routinely used in neuromonitoring for epilepsy. In 2016, Ramsey’s group implanted subdural ECoG strips over the motor cortex of a person with amyotrophic lateral sclerosis, and connected them to an IPG with real-time recording capabilities^60^. This fully implanted BMI allowed the participant to control a typing program after 28 weeks of training. In 2019, Benabid and colleagues tested a new fully implantable ECoG recording system with 64 epidural electrodes placed bilaterally over the motor cortices in a subject with tetraplegia^61^. The participant trained to control a virtual avatar and a four-limb exoskeleton, achieving up to eight DOF without any recalibration over seven weeks. Recently, Chang’s group also applied subdural ECoGs to speech neuroprostheses, decoding both full words based on a 50-word vocabulary^62^, and individual letters to produce sentences from a >1000-word vocabulary^63^. Future applications will likely require the development of novel micro-ECoG implants characterized by smaller electrodes, increased electrode densities, and flexible substrates able to conform to the cortical surface^64^.

**Fig. 1:**
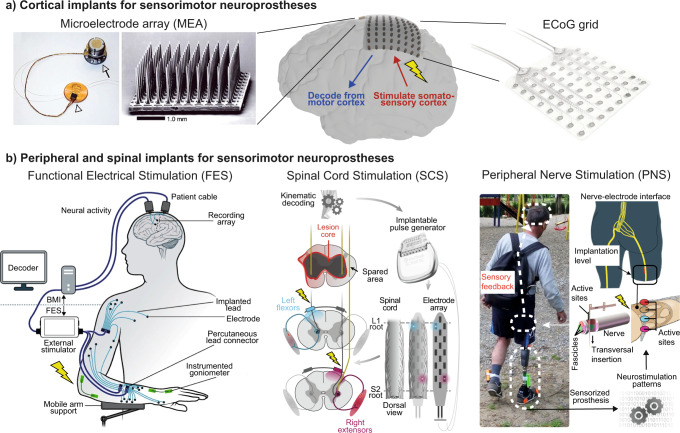
Neuroprosthetic technologies for sensorimotor disorders. **a** Cortical implants typically used for sensorimotor neuroprostheses can be divided into two categories: intracortical MEAs such as the Utah array (10 × 10 Utah array, picture extracted with permission from ref. ^55^), and epidural or subdural ECoG strips or grids with different specifications (illustrated for a 8 × 8 ECoG grid, 4 mm contact diameter, 10 mm pitch, with permission from CorTec GmbH). These implants can both record neurophysiological signals and deliver electrical stimulation. **b** Peripheral and spinal implants for sensorimotor neuroprostheses target either the motor nerves or muscles in the case of FES (adapted with permission from ref. ^66^), the spinal cord in epidural SCS (adapted with permission from ref. ^13^), or the sensory nerves in PNS for somatosensory feedback (adapted with permission from ref. ^80^). All applications shown here used these implants for delivering electrical stimulation. Lightning bolts indicate neurostimulation.

Lastly, BMIs should not be seen as a separate type of neuroprosthesis from neurostimulation methods, but rather as a potential source of control signals for operating them. Indeed, two groups showed in 2016–2017 that tetraplegic subjects with intracortical MEAs could control FES systems to achieve respectively finger, hand, and wrist movements^65^, or reaching and grasping movements involving the elbow, wrist, and hand^66^. Similarly, spatiotemporal SCS can be controlled directly by brain signals, as demonstrated in 2018 using intracortical MEAs in NHPs with a unilateral spinal cord injury^12^, and envisioned in humans using ECoG implants^67^. These various studies pave the way towards neuroprostheses that bidirectionally interface with the nervous system and harness both recording and stimulation capabilities (Fig. 1).

### Somatosensory neuroprostheses

Electrical neurostimulation can be used to induce movement as in motor neuroprostheses, but also to elicit somatic sensations such as touch or proprioception in individuals with limb amputation or paralysis^68^. In this section, we present somatosensory neuroprostheses that target either the peripheral sensory nerves of the somatosensory cortex, and the additional cognitive benefits associated with these bidirectional systems.

#### Peripheral somatosensory neuroprostheses

Peripheral somatosensory neuroprostheses, which are based on sensory PNS, have been developed to provide sensory feedback to amputees wearing a mechanical prosthesis of the missing limb. Early proof-of-concept studies by Clippinger and colleagues in the 70’s delivered stimulation through a single-channel non-penetrating cuff electrode wrapped around the median or sciatic nerve for, respectively upper- and lower-limb amputees^69,70^. Similar cuff electrodes have been used more recently in conjunction with an osseointegrated anchor to provide robust and chronic bidirectional communication with a prosthesis in upper-limb amputees over three to 7 years^71,72^. To generate more natural touch sensations, the traditional cuff electrode design has also been improved and scaled up to 16 stimulation contacts, which provided long-term natural tactile sensations in upper- and lower-limb amputees implanted for more than a year^73,74^.

The specificity of PNS depends on the ability to target specific bundles of nerve fibers, called fascicles, within a nerve. Higher specificity can therefore be achieved by electrodes that penetrate inside the nerve and reach individual fascicles. This led to the development of longitudinal intrafascicular electrodes, which were used to provide discrete and graded tactile sensations in upper-limb amputees^75–77^. Furthermore, fascicles innervating different body parts are somatotopically organized along the transverse section of a nerve, which motivated the design of transverse intrafascicular electrodes that offer higher spatial selectivity of multiple fascicles and have been applied to both the upper^78,79^ and lower extremities^80,81^. Finally, a slanted version of the Utah 96-MEA has also been used for transverse PNS and could evoke up to about a hundred different sensations in upper-limb amputees^82,83^.

#### Cortical somatosensory neuroprostheses

Somatosensory feedback can be delivered to the peripheral nerves, but also directly to the brain structures underlying somatosensation. The scientific foundations for cortical somatosensory neuroprostheses were first laid by Penfield and Boldrey back in 1937 during their mapping of sensory and motor cortices in epileptic patients^84^. These concepts were then revived in 1998 by Romo and colleagues, who demonstrated in NHPs that the sensation of mechanical flutter can be artificially induced by electrically stimulating quickly-adapting neurons in the somatosensory cortex at the frequency of the mimicked stimulus^85,86^. This observation led several other groups to show that intracortical microstimulation (ICMS) can be used as a cue to induce natural or learned behavioral responses^87–90^. Ultimately, ICMS was combined with intracortical BMIs to generate bidirectional neuroprostheses that extract motor commands from motor or association cortices and deliver feedback to the somatosensory cortex, as first demonstrated in NHPs in 2011^91,92^.

These preclinical ICMS studies paved the way for the clinical translation of cortical somatosensory neuroprostheses. In 2016 and 2018, the groups of Gaunt and Andersen implanted paralyzed individuals with chronic intracortical MEAs in the somatosensory cortex^93–97^. ICMS was shown to evoke somatotopically organized tactile sensations^93^, which included both cutaneous and proprioceptive modalities^94^. These sensations depended on stimulation frequency^95^, were stable over months and even years^96^, and improved the quality of BMI control in motor tasks^97^. In 2022, Tenore’s group refined the intraoperative mapping procedure to target specific fingertips with this technology, which allowed a participant to distinguish up to seven finger-specific locations^98^. In parallel with these chronic implantations, acute experiments in epileptic patients have demonstrated that ECoG can also be used to deliver sensory feedback by stimulating the cortical surface, but provide lower dermatome specificity than intracortical MEAs^99–102^.

#### Cognitive benefits of bidirectional sensorimotor neuroprostheses

Finally, we would like to highlight that somatosensory feedback provides not only sensorimotor but also cognitive benefits to their users as shown by the groups of Raspopovic and others^81,103–107^. These benefits include an improved embodiment of the prosthesis and reduced abnormal phantom limb perceptions in amputees^81,103,105,107^, better multisensory integration^104^, more physiological kinematics and sensorimotor strategies^106^, decreased weight perception of the prosthesis^107^ and better cognitive integration in dual tasks^81,107^. These exciting outcomes provide a strong rationale for the clinical adoption of bidirectional and biomimetic neuroprostheses that improve sensorimotor and cognitive functions after injury (Fig. 1).

### Neuromodulation for PD: from motor to cognitive symptoms

In parallel with the development of sensorimotor neuroprostheses, the electrical stimulation of deep brain structures, called DBS, was first introduced for Parkinson’s tremor by Benabid and colleagues in 1987^108^ and has since then become the main neuromodulation therapy for several motor disorders. PD is characterized by the progressive loss of dopaminergic neurons in the substantia nigra pars compacta (SNc), which results in a range of motor and non-motor symptoms. Motor symptoms include mainly tremor, limb stiffness (rigidity), slowness of movement (bradykinesia), impaired posture and balance. Non-motor symptoms include autonomic dysfunction, mood disorders, and cognitive impairment, which can range from Mild Cognitive Impairment (MCI) to Parkinson’s disease dementia (PDD) as the disease progresses^109,110^.

**Fig. 2:**
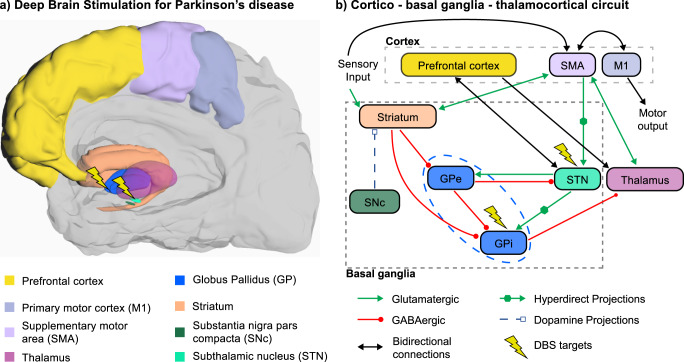
Neuromodulation targets for motor symptoms in PD. **a** Anatomical locations of the BG, cortical and thalamic areas involved in the pathophysiology of PD. DBS targeting either the STN or the GPi (lightning bolts) is one of the main treatments for motor symptoms of PD. **b** The loss of dopaminergic cells within the SNc in PD induces dysfunction of the direct and hyperdirect pathways of the cortico-BG-thalamocortical circuit, which affects both motor and cognitive functions, and can be partially restored using DBS of the STN or GPi.

PD affects the whole basal ganglia (BG) circuitry, a set of subcortical areas that comprise the subthalamic nucleus (STN), the external and internal parts of the globus pallidus (GPe, GPi), the striatum, and the SNc. These nuclei interact with cortical areas via the thalamus, thus giving rise to several parallel BG-thalamo-cortical loops (Fig. 2). The loss of dopaminergic cells in the SNc reduces dopamine levels, leading to an abnormal BG-thalamo-cortical network activity, which in turn impacts motor cortical areas involved in the generation of movement, and prefrontal areas involved in cognitive functions. Given that dopamine is also a key element in reward-based learning, these anatomical substrates explain how the symptoms of PD can affect both the motor and cognitive domains^111,112^. Here, we review the different neuromodulation targets and strategies that have been investigated to treat both motor and cognitive symptoms of PD.

#### Conventional DBS of STN or GPi for motor symptoms

The two main strategies for treating motor symptoms associated with PD are dopaminergic medication (levodopa) and DBS of the STN^113,114^ or GPi^115^, which leads in both cases to an improvement in motor symptoms and a reduction in the required levodopa medication^116^. Conventional DBS protocols, which employ frequencies in the high-gamma range (100–185 Hz), improve bradykinesia, rigidity and tremors, but are poorly effective for axial motor symptoms such as freezing of gait and swallowing. Instead, these symptoms may be better controlled by low-frequency DBS, delivered at frequencies in the lower gamma range (60–80 Hz)^117,118^.

Unfortunately, conventional DBS for PD brings little to no improvement, or sometimes even a worsening, of cognitive functions^119–121^. In particular, a meta-analysis of 41 articles gathering a total of 1622 patients reported that DBS of the STN worsens psychomotor speed, memory, semantic fluency, phenomic fluency and general cognitive functions^119^. In another study, patients who had long-term follow-up (3.6–10.5 years post-surgery) showed an overall cognitive decline and conversion from MCI to dementia, indicating that conventional DBS of the STN did not stop the cognitive decline observed in PD^120^. Although the literature on the non-motor effects of DBS of the GPi is more scarce, the cognitive outcomes seem to be slightly better when targeting GPi rather than STN^116,122^. Importantly, a recent study in 91 PD patients with bilateral DBS of the STN has shown that the precise location within the STN itself could also have an impact on the non-motor effects, including mood/apathy, attention/memory, and sleep/fatigue, which would explain the large variability observed across patients^122^.

#### The nucleus basalis of Meynert (NBM): a potential target for cognitive impairment

In addition to the loss of dopaminergic cells in the SNc, PD patients with cognitive impairment or dementia present a loss of cholinergic output from a basal forebrain nucleus called the NBM^123^. This observation has motivated several studies based on DBS of the NBM for improving cognitive function in PDD and other conditions, such as AD and dementia with Lewy bodies (DLB)^124–132^.

In an encouraging case study from 2009, a patient with PDD was implanted with four leads, placed bilaterally in the STN and NBM to treat both motor and cognitive symptoms^125^. This patient showed an improvement in cognitive functions (attention, concentration, alertness, drive and spontaneity) and changes in apraxia, when DBS was delivered simultaneously to the NBM (at 20 Hz) and STN leads, but not when stimulation was applied only to the STN^125,126^. This case study triggered a series of small-scale (six patients each) randomized, double-blind sham-controlled trials of NBM DBS at 20 Hz in patients with AD^127,133^, PD^128^, or DLB^130,134^. Although these studies validated safety and feasibility, they were rather inconclusive in terms of efficacy on cognition. Interestingly, the main outcome was an improvement in neuropsychiatric symptoms in some patients with PD or DLB, especially a reduction in visual hallucinations^128,130^.

Recently, simultaneous DBS of the NBM together with either the STN or GPi has been revisited, to treat both motor and cognitive symptoms of PD as in the original study from 2009. A case report published in 2019 showed the feasibility to target both the GPi and NBM unilaterally with a single lead, which had its most distal contact in the NBM and its second-most distal contact in the GPi^129^. Furthermore, current IPGs allow to stimulate simultaneously with different frequencies on different contacts, which was used to deliver pulses at 20 Hz and 130 Hz, respectively, in the NBM and GPi. This study showed a partial improvement in cognitive functions 3 months after the combined stimulation, which warrants further investigation.

#### Theta DBS for cognitive impairment in PD

Motor and cognitive symptoms of PD may be treated by targeting specific anatomical structures with DBS, but also by applying different stimulation frequencies to a given structure, which may in turn recruit different network dynamics. In particular, there is ample evidence for the role of theta oscillations (4–12 Hz) in cognitive processes. This observation has led several groups to compare the cognitive effects of STN DBS in the theta range (at either 5 or 10 Hz) with more standard protocols in the high-gamma range (130 Hz)^135–138^.

A first study in 2006 reported that 10-Hz stimulation of the STN significantly improved verbal fluency compared to standard 130-Hz stimulation in 12 patients with PD^135^. There was also a non-significant trend towards improvement when compared to the absence of stimulation. A later study in 15 subjects showed that 5-Hz DBS of the STN significantly improved performance on verbal processing speed and response inhibition through color-word interference, when compared to no stimulation and 130-Hz DBS^136^. Recently, two additional studies revisited these concepts. One clinical trial in 12 patients systematically assessed verbal fluency for episodic and non-episodic categories, color-word interference, and random number generation, during DBS of the STN at 130 Hz, at 10 Hz, or in the absence of stimulation^137^. The results indicated a significant improvement specifically for the episodic verbal fluency task when theta DBS was delivered, an interesting finding given the prominence of this rhythm in episodic memory. Finally, the latest study investigated the impact of stimulation location (ventral vs dorsal part of the STN) and frequency (personalized theta vs 130 Hz) on verbal fluency in nine patients^138^. Theta DBS was delivered at frequencies between 4 and 8 Hz depending on the subject (mean 5.7 Hz). Importantly, it was found that theta DBS of the dorsal part of the STN yielded significantly better cognitive outcomes than no stimulation or gamma stimulation of either the dorsal or ventral part of the STN.

### Neural oscillations underlying cognition in health and memory disorders

In the previous section, we reviewed how neuromodulation strategies originally designed for motor symptoms could be adapted to cognitive symptoms in the context of PD, by either targeting different anatomical targets or tapping into different networks based on the stimulation frequency. Hereafter, we focus on the role of neuronal oscillations in cognitive processes in general, and memory in particular, with the intent to develop neuromodulation strategies for neuronal disorders that are primarily cognitive by nature, such as memory impairments and AD.

#### Neural oscillations in normal cognitive functions

Cognitive functions are associated with neural oscillations in specific frequency ranges affecting higher order neocortical areas, called association cortices, and other limbic or subcortical structures such as the hippocampus, especially in the theta (4–12 Hz) and gamma (~40 Hz) ranges^139^. These oscillations usually affect simultaneously multiple brain structures, which belong to a common large-scale brain network subserving a given cognitive function^140,141^. It is thought that long-range phase synchronization in the low frequencies (especially theta) allows remote areas to communicate with each other^142–144^. Moreover, coupling between oscillations in two different frequency bands, known as cross-frequency coupling (CFC), allows these long-range interactions to influence local gamma oscillations and information processing. The most widely studied example is the coupling between the phase of theta oscillations and the amplitude of gamma oscillations in limbic circuits^145–152^. Together, long-range phase synchronization and both local and long-range CFC allow complex interactions between remote areas belonging to the same large-scale brain network^153^. Understanding these interactions, how they are affected in cognitive disorders, and how to restore them using neuromodulation, is therefore critical for the development of future therapies^15,154^.

**Fig. 3:**
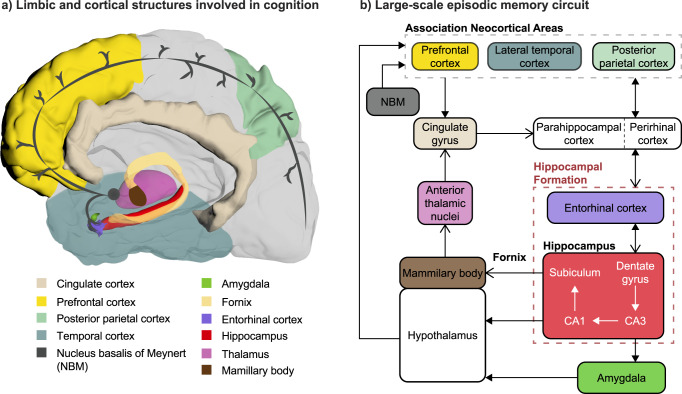
Possible neuromodulation strategies for cognitive impairment and dementia. **a** Limbic and cortical structures involved in cognition, in particular episodic memory. These structures are part of a common large-scale brain network. **b** Schematic representation of the connections between these structures, including intra-hippocampal circuitry (red dashed box), the original circuit of Papez (open arrowheads), and additional connections between them (filled arrowheads). The NBM provides widespread cholinergic innervation to neocortical areas and has therefore received attention as a neuromodulation target for cognition. The fornix has been the main chronic DBS target in AD patients. Other areas (hippocampus, EC, neocortical areas) have been mainly targeted in acute studies in epileptic patients.

Among all cognitive processes, learning and memory have received considerable research attention because of their pivotal role in our daily lives. We will focus here on the circuit underlying episodic memory (Fig. 3), which is a form of long-term memory underlying the learning and recollection of past events. Episodic memory involves bidirectional interactions between the hippocampus and other cortical areas, including the prefrontal cortex (PFC)^155,156^. These interactions are partly mediated by the entorhinal cortex (EC), which acts as a hub between the hippocampus and these cortical areas. The dorsolateral part of the PFC (dlPFC) is thought to be mostly associated with working memory, or the maintenance of information (e.g., from sensory cortices) for a short time period before being used by other cognitive processes. The medial PFC (mPFC) is involved in memory consolidation and provides contextual information during episodic memory retrieval. In addition to the connections mediated by the EC, there is a direct projection from the ventral/anterior hippocampus to mPFC, which may be associated with this role in processing contextual information.

In summary, there is strong evidence that functional interactions between the hippocampus and PFC involve theta oscillations in both structures, theta phase synchronization, and theta-gamma CFC^142–152^. Recent evidence in NHPs shows that other oscillations such as alpha and beta in the hippocampus and PFC are also involved in associative learning^157^. Overall, these oscillations seem to be not just an epiphenomenon, but to play a causative role in efficient memory encoding and retrieval^6^.

#### Alteration of network connectivity and neural oscillations in AD and memory disorders

Memory disorders occur when a part of this episodic memory circuit gets disrupted. This finding has been supported by a large body of evidence showing structural and functional disconnections between nodes of the large-scale episodic memory network in neurodegenerative diseases^158,159^, post-traumatic amnesia^160^ and other forms of memory disorders^161^. For example, the EC is one of the first areas to be affected in the early stages of AD, even before the first symptoms are diagnosed, and plays a key role in the communication between neocortical areas and the hippocampus^162^. In post-traumatic amnesia, mechanical damage to the long-range white matter tracts connecting these structures also mediates the associated deficits^160^.

This structural and functional disconnection results in an alteration of neural oscillations and impacts cognitive processes. Such alterations in theta and gamma rhythms and their CFC have been observed in AD and temporal lobe epilepsy^161^. A long history of EEG studies in patients with AD has shown an increase in low-frequency delta and theta rhythms, and a decrease in faster alpha and beta rhythms^163^. Some studies have also reported either a decrease^164^ or an increase^165,166^ in gamma oscillations, a change in the modulation of theta during a working memory task in subjects with AD or MCI^167^, and either a reduced^165^ or a larger^168,169^ theta-gamma CFC. Overall, the observed changes are not always consistent from one clinical study to another and may depend on the recording condition (resting-state, steady-state evoked potentials, or during a cognitive task), on interindividual variability, and on potential compensatory mechanisms that cloud the interpretation of these reports.

Despite the variability and relative scarcity of human studies on this topic, the changes in theta and gamma rhythms, as well as their CFC, have also been thoroughly investigated in animal models of AD. The results in individual theta and gamma rhythms were not quite conclusive in these models or did not mirror the observations in patients^161^, but a reduced theta-gamma CFC was consistently observed in several transgenic mouse models of AD^159,170–172^. Only one study has found an opposite trend in a rat model of hyperglycemia, which is a risk factor for AD^173^. Although more work is needed in different animal models and at different timepoints during the evolution of each phenotype, theta-gamma CFC appears as the most reproducible oscillatory alteration both in animal models and in humans with AD. This observation is quite compelling given the role of CFC in non-pathophysiological learning and memory processes.

### Neuromodulation for AD and memory disorders

The observation that neural oscillations play a key role in cognitive processes and are disrupted in a range of memory disorders, including AD, leads us to the enticing hypothesis that restoring these oscillations and their couplings might rescue the associated symptoms^15,154^. In turn, chronic restoration of these oscillations might trigger neuroplasticity mechanisms, slow down the progression of neurodegenerative processes, or even lead to long-lasting improvements^174^. Below, we review the main intracranial neuromodulation studies that have aimed at improving cognition and memory in patients with AD chronically implanted with DBS electrodes, or in epileptic patients who underwent acute intracranial monitoring prior to resective surgery (Table 1).

**Table 1:** Intracranial neuromodulation studies with encouraging outcomes for improving cognition or memory in PD, AD and epilepsy patients.

Main target	Condition	Number of subjects	Summary	Reference
STN	PD	12	Verbal fluency improved with 10-Hz DBS compared to 130-Hz DBS	Wojtecki et al.^135^
STN	PD	15	Verbal processing speed/response inhibition improved with 5-Hz (but not 130-Hz) DBS compared to no DBS	Scangos et al.^136^
STN	PD	12	Episodic verbal fluency improved with 10-Hz DBS compared to 130-Hz DBS, but not compared to no DBS	Lam et al.^137^
STN	PD	9	Episodic verbal fluency improved with theta-DBS (4–8 Hz, personalized) of dorsal STN, compared to no DBS and to 130-Hz DBS of dorsal/ventral STN	Lee et al.^138^
NBM	AD	1	DBS for 9 months increased glucose metabolic activity in the ipsilateral temporal and parietal lobes but had no clinical effect on cognition or memory	Turnbull et al.^124^
NBM + STN	PD	1	Improvement in attention, concentration, alertness, drive and spontaneity during DBS of NBM (20 Hz) and STN, but not during STN DBS only	Freund et al.^125^
NBM + STN	PD	1	Changes in apraxia during DBS of NBM (20 Hz) and STN, but not during STN DBS only	Barnikol et al.^126^
NBM	AD	6	Bilateral DBS (20 Hz) led to stable or improved cognitive function 12 months after surgery in four out of six patients with mild to moderate AD	Kuhn et al.^127^
NBM	AD	2	Two patients at an earlier phase of AD showed stable memory function after 2 years of DBS	Kuhn et al.^133^
NBM	PD	6	Bilateral DBS (20 Hz) did not improve cognition but decreased visual hallucinations in some patients	Gratwicke et al.^128^
NBM + GPi	PD	1	Combined 130-Hz GPi DBS and 20-Hz NBM DBS partially improved both motor and cognitive functions	Nombela et al.^129^
fornix	morbid obesity	1	DBS increased autobiographical memory recollection but not familiarity-based recognition	Hamani et al.^176^
fornix	AD	6	DBS drove neural activity in the memory circuit, increased cortical glucose metabolism, and led to stable or improved cognitive function in some patients	Laxton et al.^177^
fornix	AD	1	Increase in mesial temporal lobes metabolism and stabilization of memory scores after 1 year of DBS	Fontaine et al.^178^
fornix	epilepsy	4	Theta-burst DBS improved visuospatial memory, but led to inconsistent results on verbal memory or naming	Miller et al.^185^
fornix	AD	6	Hippocampal volume increased bilaterally after 1 year in two patients, including one with improved cognition	Sankar et al.^179^
fornix	AD	42	Increased cerebral glucose metabolism after 6 months but no significant effect on cognitive functions	Lozano et al.^180^
fornix	AD	42	Trend towards a delay in cognitive decline in older patients (>65-year old) compared to younger subjects	Leoutsakos et al.^181^
EC	epilepsy	7	DBS of EC but not HC applied during spatial memory encoding enhanced subsequent retrieval	Suthana et al.^190^
EC + HC	epilepsy	11	Trend towards improvement of verbal memory encoding during in-phase DBS of EC and HC compared to sham or anti-phase stimulation	Fell et al.^197^
EC	epilepsy	13	Theta-burst microstimulation of the right entorhinal area during learning improved face recognition memory	Titiz et al.^192^
HC^a^	epilepsy	22	Closed-loop microstimulation of CA1 from CA3 spiking activity during encoding improved working memory	Hampson et al.^198^
amygdala	epilepsy	14	Brief (1 s) stimulation increased declarative memory without eliciting a subjective emotional response	Inman et al.^195^
HC	epilepsy	6	DBS during encoding improved associative memory and increased theta power in successful trials	Jun et al.^194^
EC	epilepsy	22	DBS of right entorhinal white matter enhances learning, unlike grey matter or left-sided stimulation	Mankin et al.^193^
HC^a^	epilepsy	22	Closed-loop CA1 microstimulation is more efficient in patients with brain injury and/or memory impairments	Roeder et al. ^208^
many areas	epilepsy	27	Stimulation delivered during bad encoding improved retrieval, the opposite effect observed during good encoding	Ezzyat et al.^202^
temporal cortex^a^	epilepsy	25	Closed-loop stimulation of lateral temporal cortex in bad encoding trials improves memory performance	Ezzyat et al.^203^
temporal cortex	epilepsy	22	Stimulation of lateral temporal cortex (but not other areas) during encoding enhanced verbal memory recall	Kucewicz et al.^200^
temporal cortex	epilepsy	22	Stimulation that improved memory also increased high-gamma power induced by word presentation in encoding	Kucewicz et al.^201^
frontal + parietal cortices	epilepsy	3	Stimulation of frontal and parietal cortices in-phase within theta or alpha range improved working memory, compared to sham or anti-phase stimulation	Alagapan et al.^205^

*HC* hippocampus.

^a^Closed-loop stimulation.

#### DBS of the NBM in AD

Historically, the NBM was the first DBS target to be investigated as a potential symptomatic treatment of AD. As discussed previously, this structure has been targeted in PDD, AD and DLB to increase the release of acetylcholine, which led to improved neuropsychiatric outcomes in some patients^131^. In the context of AD, an original case study from 1985 showed increased glucose metabolic activity in the ipsilateral temporal and parietal lobes but no effect on cognition or memory^124^. More recently, bilateral DBS of the NBM led to stable or improved cognitive function 12 months after surgery in four out of six patients with mild to moderate AD^127^. In a follow-up study, the same procedure was applied to two patients at an earlier phase of the disease^133^. In both of them, overall cognitive function was improved or stable during the first year of DBS and either returned to baseline or degraded during the following year, while memory function was stable over these 2 years. Additionally, clock agnosia completely disappeared in one patient. Although inconclusive due to the small number of subjects, these results motivate future work and possible refinements to modulate cholinergic brain networks in AD. For example, intermittent stimulation of the NBM (20 s every minute) has been shown to improve working memory in NHPs, whereas continuous stimulation impaired memory^175^.

#### DBS of the fornix in AD and epileptic patients

The fornix is a bundle of fibers that contains both the main output tract of the hippocampus and some afferent fibers from areas such as the basal forebrain. Because its integrity is critical to episodic memory, it appears as a target of choice for modulating memory function. In 2008, Lozano and colleagues made the serendipitous observation that DBS of the fornix evoked vivid recollection of memories in a patient treated with hypothalamic DBS for obesity^176^. This prompted them to perform a phase I safety and feasibility study of fornix DBS in six patients with mild AD^177^. DBS was delivered continuously, with stimulation amplitudes lower than those used to evoke vivid memories. Positron Emission Tomography (PET) revealed that glucose metabolism increased after both 1 month and 1 year of stimulation, especially in the temporal and parietal cortices. Some patients showed stable or improved cognitive or memory functions at six and/or 12 months post-surgery, but the results were not consistent across subjects and varied with the type of assessment. Similar results were later reported in one patient with mild AD by an independent group^178^.

In a subsequent report, the authors investigated the volume change of the hippocampus, mammillary bodies, and fornix following 1 year of stimulation in the same six patients^179^. Unlike the expected hippocampal atrophy normally seen in AD patients, an increased hippocampal volume was observed bilaterally in two of the patients, including the one who showed improved cognitive performance after one year. By contrast, bilateral hippocampal volume increase was never observed among the 25 matched subjects from the AD Neuroimaging Initiative database^179^.

In a follow-up double-blinded phase II clinical trial, DBS was applied bilaterally to the fornix of 42 patients with mild AD^180,181^. Half of the patients received DBS immediately after implantation, while the other half only received DBS after 1 year of sham stimulation (i.e. with the stimulator turned off). Fornix DBS significantly increased cerebral glucose metabolism after 6 months in the group receiving stimulation, but it did not reveal any statistically significant effect on cognitive functions. Post-hoc analyses indicated a non-significant trend towards cognitive benefits in older patients (>65-year old), which may be due to the fact that younger patients are usually affected by more malignant forms of the disease.

In all the above studies, stimulation was applied at 130 Hz as commonly used for PD. However, several animal studies have investigated other frequencies and patterns of fornix DBS, in particular theta and theta-burst stimulation^182,183^. Theta-burst stimulation consists of high-frequency bursts (typically 4–20 pulses at 100–500 Hz), nested within a low-frequency envelope in the theta range (4–8 Hz). It is optimal for inducing Long-Term Potentiation (LTP) in the hippocampus in vitro^184^, and superior to both conventional high-frequency stimulation (130 Hz) and low-frequency theta stimulation for improving learning and memory in a rat pharmacological model of amnesia^182^ and a rat model of traumatic brain injury^183^. Based on these results, one clinical study tested the immediate effects on memory of theta-burst DBS of the fornix in four epileptic patients^185^. They showed an immediate and reversible improvement in a visuospatial memory task in all four patients, but inconsistent results in other tasks related to verbal memory or naming.

#### DBS of the entorhinal area and other limbic structures in epileptic patients

Because of its major role in learning and memory, the hippocampus appears as an obvious neuromodulation target for memory enhancement. However, direct electrical stimulation of the hippocampus was shown to impair memory processes in early reports from 1985 that used amplitudes above the threshold to elicit epileptiform afterdischarges^186,187^. More recently, a similar impairment was also shown with stimulation amplitudes below the afterdischarge threshold^188,189^.

Instead of the hippocampus itself, its afferents from the EC could also be stimulated. In a first pivotal study in seven epileptic patients, Suthana and colleagues showed that DBS of the EC during spatial memory encoding improved subsequent retrieval at the group level^190^. The same stimulation applied to the hippocampus did not reveal any significant effect. However, in an attempt to replicate these results, Jacobs and colleagues found that DBS of either the EC or the hippocampus during spatial and verbal memory encoding actually disrupted subsequent retrieval^191^, probably due to differences in the exact DBS location between the two studies. In follow-up work, theta-burst microstimulation, known to optimally induce hippocampal LTP, was delivered to the right EC through thin microelectrodes during memory encoding, resulting in improved performance on a face recognition task^192^. The same group also compared the effects of stimulating the grey versus white matter of the entorhinal area, the lateralization of these effects, and the impact of electrode size (macro- versus microelectrode) in 22 epileptic patients^193^. Importantly, they identified the right entorhinal white matter (so-called angular bundle) as the optimal stimulation site to improve memory encoding. By contrast, electrode size was not a significant predictor of memory performance.

Further research is still needed to ask whether these results are robust across subjects and experimental conditions. For example, one study showed improvement in memory encoding during direct hippocampal stimulation, and this discrepancy was attributed to differences in the memory tasks^194^. Memory enhancement or disruption could also be achieved by stimulating other targets within the limbic circuit, such as the amygdala^195^ or the posterior cingulate cortex^196^. Finally, other approaches such as multisite synchronized^197^ and/or closed-loop stimulation^198^ within the limbic circuit could be used to refine these protocols, as detailed in the last paragraph of this section.

#### Direct cortical stimulation of neocortical areas in epileptic patients

In parallel with DBS of limbic structures, the effects of neocortical stimulation on memory performance have also been investigated. The earliest report that temporal lobe stimulation can evoke visual and auditory experience of past memories dates back to Penfield and Perot in 1963^199^. Recently, a series of collaborative studies led by Worrell and Kahana, respectively, at the Mayo Clinic and the University of Pennsylvania revisited this concept^200–203^.

Kucewicz and colleagues investigated the effects of electrical stimulation of four structures (hippocampus, parahippocampal neocortex, PFC and temporal cortex) in 22 patients with epilepsy (each structure was stimulated in only a subset of the patients)^200,201^. Stimulation was delivered during the encoding phase of a verbal memory task using depth electrodes and subdural ECoG grids. They found that only stimulation of the lateral temporal cortex was able to enhance subsequent retrieval, which was observed both at the group level and in two of the four tested participants^200^. Electrophysiologically, word presentation induced high-gamma activity (62-118 Hz) in several areas. Stimulation caused an increase of this induced activity in the lateral temporal cortex, which was associated with memory enhancement, but a decrease in the other brain areas. Therefore, high-gamma activity could be a reliable biomarker for predicting stimulation effects^201^.

Additionally, Ezzyat and colleagues trained machine learning algorithms to discriminate between trials of good and bad encoding based on neural activity preceding the stimulation period^202^. They showed that stimulation delivered during trials of bad encoding improved memory performance, while stimulation during good encoding trials decreased performance. This dependency on the encoding state was deemed to account for the variability observed in the effects of stimulation across patients and trials.

#### Closed-loop and multi-site approaches for intracranial neuromodulation of memory

All results described so far employed open-loop stimulation delivered either continuously or applied during memory encoding to improve subsequent retrieval. Such protocols do not take into account the ongoing activity of the implanted brain areas, which is influenced not only by the stimulation but also by intrinsic changes in neural activity. To mitigate this limitation, the patterns and parameters of stimulation could be adjusted in real-time based on the current neural state, a strategy called closed-loop stimulation^198,203,204^.

A closed-loop approach was demonstrated by Ezzyat and colleagues following their discovery that stimulation effects depend on the ongoing neural state. Using multivariate classifiers able to classify trials with good or bad encoding^202^, they delivered stimulation to the lateral temporal cortex only when a trial of bad encoding was detected^203^. This protocol improved retrieval in a more consistent way than open-loop stimulation.

To further refine these techniques, neurostimulation could also be applied to multiple sites, corresponding to different nodes of the distributed memory network, with the appropriate phase relationship to mimic physiological processes. In this spirit, Alagapan and colleagues applied network-targeted stimulation, which was delivered at two cortical locations (frontal and parietal) either in-phase or in anti-phase, within the theta or alpha range depending on the participant^205^. Tested in three epileptic patients during a working memory task, this strategy affected phase synchronizations within the targeted network and improved working memory.

Finally, the most versatile neurotechnologies should probably include both aspects: closed-loop and multisite stimulation. A memory neuroprosthesis based on these concepts was pioneered by Berger and colleagues in rats^206^, later tested in NHPs^207^, and recently in epileptic patients^198,208^. The authors extracted neuronal signals from microelectrodes implanted into the CA1 and CA3 fields of the hippocampus to build a multi-input multi-output non-linear model able to predict the spiking activity of CA1 neurons based on the activity of CA3 neurons. This model was then used to control spatiotemporal patterns of electrical stimulation delivered to the CA1 field based on real-time recordings from CA3 neurons. Stimulation applied during the encoding phase of a working memory task led to improved performance. Importantly, the effect in epileptic patients was more pronounced in those having a prior history of brain injury and memory impairments, demonstrating the suitability of this approach for memory disorders^208^. This conceptually very interesting approach would be suitable when memory impairments originate specifically from dysfunction within the hippocampus, but does not target the overall large-scale brain networks involved in episodic memory.

### Outlook: towards large-scale memory neuroprostheses

#### The need for novel neurostimulation strategies

Considering that episodic memory relies on a distributed network of brain areas that interact via long-range connections through phase synchronization mechanisms and phase-amplitude CFC, we hypothesize that a physiological stimulation paradigm for memory enhancement or restoration should include the following key features:

• *Spatial specificity*: different areas (hippocampus, EC, PFC, etc.) should be targeted independently by the stimulation.

• *Spectral specificity*: each area should be stimulated with a particular frequency or a set of superimposed frequencies (e.g. theta to promote long-range interactions and gamma for local information processing).

• *Temporal specificity*: this aspect can be divided into two different timescales. On a long timescale (on the order of hundreds of milliseconds to seconds), the pattern of brain activation should depend on the task being carried out (e.g. encoding or retrieval) and potentially on the stage within this task. On a short timescale (tens of milliseconds), the phase of each stimulation pulse within each area should be carefully adjusted so that different areas are stimulated with the appropriate phase relationship (in-phase, out-of-phase, etc.).

**Fig. 4:**
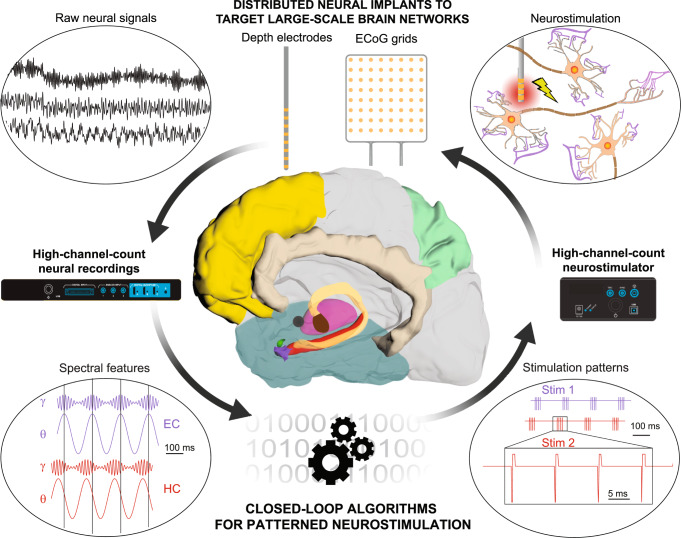
Towards large-scale bidirectional neuroprostheses for memory disorders. Future neuroprosthetic systems for memory restoration may benefit from distributed multi-electrode implants targeting large-scale brain networks, combining depth electrodes, ECoG grids and potentially novel electrode technologies for combined recordings and stimulation. Field potentials measured by these implants would then be sent to computing devices via high-channel-count recording systems. These computing devices would extract in real-time specific spectral features of these raw neural signals, such as the instantaneous power, phase and phase-amplitude coupling of theta and gamma oscillations in hippocampal structures. These features would then be used as control signals in closed-loop algorithms that deliver spatially, temporally and spectrally patterned neurostimulation protocols to enhance the neural features of successful memory processes (encoding or retrieval). These complex stimulation patterns would next be applied to the distributed neural implants via high-channel-count neurostimulators. Neurostimulation would finally affect neuronal activity through mechanisms that depend on the geometry of local fibers, which can be explained or theoretically predicted by computational models of neurostimulation. Schematics of high-channel-count recording and stimulation systems were adapted with permission from Blackrock Neurotech.

These different features require a technological framework for spatially, temporally, and spectrally patterned neuromodulation of the large-scale episodic memory network to improve memory encoding and retrieval (Fig. 4), which critically depends on two key elements:

• the ability to simultaneously record and stimulate the large-scale episodic memory network through novel neural implants that target distributed brain areas, for example through intracerebral depth macroelectrodes classically used for stereo-encephalography (stereo-EEG), and subdural ECoG implants.

• the development of novel neurostimulation strategies that promote physiological oscillations throughout this distributed network, which require patterned stimulation protocols (such as theta burst) and the ability to trigger them (responsive stimulation) or adjust their stimulation parameters in real-time based on ongoing brain activity (adaptive or closed-loop stimulation).

#### Novel electrode technologies: opportunities and limitations

Both depth macroelectrodes and ECoG grids used in epileptic patients take their roots back to the middle of the 20th century^209–211^. Although clinically validated, these adopted neurotechnologies suffer from several impediments, namely the relatively small number of channels and large recording/stimulation volumes, which may limit the spatial selectivity required for the fine manipulation of cognitive and memory functions. Cognitive neuroprostheses might therefore benefit from more refined electrode technologies that can target both deep and cortical structures in large-scale brain networks at multiple spatial scales^9^.

For example, recent advances in micro- and nano-fabrication technologies have led to the development of silicon probes, which enable the simultaneous recording of tens and even thousands of neurons deep inside the brain^212–223^. These ultra-thin shanked probes (cross section: 20–120 μm, length: up to 20 mm) allow simultaneous recordings from up to 1356 sites^224^. Some probes (Neurotech Alliance, E-beam, Silicon microprobes, etc.) are designed with multiple shanks for greater cortical and subcortical area coverage. For single-shank probes such as Neuropixels, larger brain areas can be targeted using multiple probes, a strategy that allowed to record from approximately 30,000 neurons located in 42 regions in the mouse brain^224,225^.

The use of such high-density multi-electrode and multi-shank probes presents both advantages and challenges. Compared to larger conventional electrodes, these much smaller silicon probes have reduced neural tract damage. However, the ultra-small cross-sectional area and stiffness may lead to electrode drift due to blood flow, and make breakages frequent^212,215^. Like conventional electrodes, silicon probes also show inflammatory response and tissue scarring that deteriorates the quality of signals over time^212^, but recordings up to 3 months are feasible^226^. To mitigate tissue scarring and electrode drifts, a dissolvable shaft has also been developed^212^. Notably, tissue response was not observed until about 1 year for some probes^216–218^, while for others the duration was as short as 2–3 weeks^226^.

Most of these studies have been focused on rodents, with only a few reports in NHPs^223,227^ and humans undergoing intraoperative neurosurgical procedures^219,228^. Challenges associated with these early clinical demonstrations include probe breakage and recording of fewer neurons, due to the short experimental duration (<1 h) and the time necessary for neuronal activity to stabilize. An additional limitation is that Neuropixels probes are currently not suitable for neurostimulation^224^. Despite these current challenges, they represent an attractive option for the next-generation neuroprostheses targeting large-scale brain networks.

#### The use of NHPs in cognitive neuroprosthetic research

To allow the translation of cognitive neuroprosthetic technologies into clinical practice, and given the regulatory constraints on the development of novel medical devices, we believe that large animal models are required. Because of their similarity in anatomy, functional connections and default-mode network topology with the human brain^229,230^, NHPs appear as the best species for investigating large-scale network dynamics and developing invasive neuromodulation strategies that should eventually be used in humans.

Like humans, NHPs develop task-specific cognitive impairments associated with normal ageing, in particular in the fields of visuospatial or spatial working memory, which depend on the PFC alone or in interaction with hippocampal-temporal structures^231–233^. These deficits mainly mimic normal cognitive ageing and are associated with amyloid-beta senile plaques. In addition to natural ageing, memory deficits can be reversibly induced by pharmacological agents such as the anticholinergic drug scopolamine, which has been shown to impair working memory in both rhesus and cynomolgus macaques during various tasks^234–236^.

Until recently, studies had failed to find other signs of AD, such as tau pathology, neurofibrillary tangles, or behavioral symptoms in most NHP species^237,238^, except in the mouse lemur *Microcebus murinus*^239^. However, this picture has recently changed, with several groups finding evidence of tau pathology similar to human AD in some (but not all) of aged (more than 20 years) African green monkeys^240,241^, rhesus^242,243^ and cynomolgus macaques^244^. To artificially replicate the pathophysiological features of AD in NHP, several strategies have also been tested such as the induction of insulin deficiency using streptozotocin^245,246^, the intraventricular injection of synthetic amyloid-beta oligomers^247^, or the injection into the EC of a viral vector expressing a double tau mutation^248^. Finally, the advent of genetic engineering tools in NHPs has recently led to the generation of marmoset monkeys carrying mutations in the PSEN1 gene, which is involved in familial forms of AD^249,250^. However, none of these NHP models of AD has been validated at a behavioral level, which is essential for testing the efficacy of cognitive neuroprostheses.

#### Computational models of neurostimulation in the context of memory processes

Computational modeling has been extensively used to better understand the effects of electrical neurostimulation, ranging from DBS in PD, to PNS, SCS, cochlear implants, and retinal prostheses (e.g.^251^). However, in the context of memory, even state-of-the-art computational models are limited in their ability to integrate hippocampal oscillations, memory processes, and the effects of electrical stimulation on local circuits and distant connected areas in large-scale brain networks. From a neuroengineering viewpoint, there is therefore an urgent need to develop novel biologically realistic computational models that guide electrode design, placement and stimulation protocols to enhance the efficacy of memory neuroprostheses^252,253^.

#### Computational models of theta-gamma neural oscillations and memory processes

Neural oscillations such as theta, gamma and their CFC have been modeled at different levels of resolution, from abstract neural masses to more biophysically realistic conductance-based models. Neural masses comprising two interconnected populations of excitatory and inhibitory neurons can produce gamma oscillations in the presence of an external input provided at a theta frequency, hence leading the theta-nested gamma oscillations^254,255^. Such oscillations also emerge from a conductance-based model of the hippocampal formation containing single-compartment excitatory and inhibitory neurons in the EC, dentate gyrus, CA3 and CA1 fields of the hippocampus, still in the presence of an external theta input^256^. Theta-gamma CFC also appears in a conductance-based multicompartment model of neocortex, which includes two cortical layers, a hypercolumn and minicolumn structure, and a built-in attractor network that represents the reactivation of specific cell assemblies during memory retrieval^257,258^.

Linking these oscillations with memory processes represents another major challenge. One such attempt explored the biophysical mechanisms of memory encoding and retrieval in a multicompartment model of the hippocampal CA1 field, demonstrating that the modulation of dendritic and somatic inhibition by the theta rhythm influences LTP and cell output^259^. Furthermore, another multicompartment study of the hippocampal CA3 showed that cholinergic deprivation leads to a slowing of gamma oscillations produced by the network, which can potentially affect the ability to store and recall information accurately^260^. These works provide the necessary leverage to investigate how neural oscillations and memory processes are altered in neurological disorders such as AD.

#### Computational models of extracellular electrical neurostimulation

Most models of electrical stimulation have focused on the mechanisms underlying DBS in motor disorders such as PD (e.g. refs. ^261–264^), often approximating stimulation as an intracellular current that enters into the soma. However, the effects of extracellular electrical stimulation are much more complex, as illustrated by a series of modeling studies that date back to the 80s^265–269^. McNeal and Rattay initially introduced cable models to describe axons as a series of capacitance and resistance circuits, similar to the Hodgkin-Huxley formalism but as a cable following the axonal trajectory^265,266^. They found that the level of activation of nerve compartments depends on the second spatial derivative of the electrical potential along the membrane. Subsequent studies investigated in more detail the effects of tissue properties^268^, excitation thresholds^269^ and action potential propagation^267^. Cable models successfully explained the mechanisms underlying various neuroprostheses^251^ and reconciled contradictory hypotheses on the effects of DBS in PD^270^.

Despite the wealth of computational work that involves neurostimulation of peripheral nerves or BG, very few studies have attempted to replicate hippocampal stimulation in a biologically realistic way. A detailed model of the dentate gyrus, which involves granule cells and entorhinal afferents^271^, was confronted with experimental data during electrical stimulation in rat hippocampal slices^252^. This study demonstrated the ability to predict the optimal electrode placement to maximize population response within localized structures. However, models that integrate more widespread brain areas in a biologically realistic way are still lacking.

Recently, a neuroimaging and computational pipeline for creating personalized computational models of the spinal cord was developed to predict the effects of spinal cord stimulation protocols on the recruitment of afferent fibers in lumbosacral spinal segments^14^. This pipeline guided the design of new electrode arrays and their accurate placement during surgery. We envision that a similar approach combining neuroimaging and computational modeling can be used to create personalized models of the hippocampal formation and predict the effects of electrical stimulation at various locations within this structure and with different stimulation parameters and control protocols.

### Conclusion

Since their very first prototypes about half a century ago, sensorimotor neuroprostheses have steadily evolved, paralleling progress in neurophysiology and engineering sciences. While some of these technologies are already clinically accepted, others are still making their way through the hurdles of clinical and regulatory validation, from preclinical and pilot studies to multicentric clinical trials. Neuromodulation and neuroprosthetic technologies have also received considerable attention from the scientific community for their potential in treating cognitive disorders. Recent exploratory studies open exciting avenues for the future development of such neuroprosthetic systems that affect neural oscillations throughout large-scale brain networks, palliate the associated deficits and potentially trigger neuroplasticity. We believe that these endeavors will require the elaboration of novel neural implants and neurostimulation protocols, the use of appropriate preclinical models to test them, and will be refined by computational approaches derived from neuroimaging data.
